# The Chilling Effect: How Do Researchers React to Controversy?

**DOI:** 10.1371/journal.pmed.0050222

**Published:** 2008-11-18

**Authors:** Joanna Kempner

**Affiliations:** Rutgers University, Department of Sociology and Institute for Health, Health Care Policy and Aging Research, New Brunswick, New Jersey, United States of America; University of Toronto, Canada

## Abstract

**Background:**

Can political controversy have a “chilling effect” on the production of new science? This is a timely concern, given how often American politicians are accused of undermining science for political purposes. Yet little is known about how scientists react to these kinds of controversies.

**Methods and Findings:**

Drawing on interview (*n* = 30) and survey data (*n* = 82), this study examines the reactions of scientists whose National Institutes of Health (NIH)-funded grants were implicated in a highly publicized political controversy. Critics charged that these grants were “a waste of taxpayer money.” The NIH defended each grant and no funding was rescinded. Nevertheless, this study finds that many of the scientists whose grants were criticized now engage in self-censorship. About half of the sample said that they now remove potentially controversial words from their grant and a quarter reported eliminating entire topics from their research agendas. Four researchers reportedly chose to move into more secure positions entirely, either outside academia or in jobs that guaranteed salaries. About 10% of the group reported that this controversy strengthened their commitment to complete their research and disseminate it widely.

**Conclusions:**

These findings provide evidence that political controversies can shape what scientists choose to study. Debates about the politics of science usually focus on the direct suppression, distortion, and manipulation of scientific results. This study suggests that scholars must also examine how scientists may self-censor in response to political events.

## Abstract 2

Drawing on interview and survey data, Joanna Kempner's study finds that political controversies shape what many scientists choose not to study.

## Editors' Summary

**Background.:**

Scientific research is an expensive business and, inevitably, the organizations that fund this research—governments, charities, and industry—play an important role in determining the directions that this research takes. Funding bodies can have both positive and negative effects on the acquisition of scientific knowledge. They can pump money into topical areas such as the human genome project. Alternatively, by withholding funding, they can discourage some types of research. So, for example, US federal funds cannot be used to support many aspects of human stem cell research. “Self-censoring” by scientists can also have a negative effect on scientific progress. That is, some scientists may decide to avoid areas of research in which there are many regulatory requirements, political pressure, or in which there is substantial pressure from advocacy groups. A good example of this last type of self-censoring is the withdrawal of many scientists from research that involves certain animal models, like primates, because of animal rights activists.

**Why Was This Study Done?:**

Some people think that political controversy might also encourage scientists to avoid some areas of scientific inquiry, but no studies have formally investigated this possibility. Could political arguments about the value of certain types of research influence the questions that scientists pursue? An argument of this sort occurred in the US in 2003 when Patrick Toomey, who was then a Republican Congressional Representative, argued that National Institutes of Health (NIH) grants supporting research into certain aspects of sexual behavior were “much less worthy of taxpayer funding” than research on “devastating diseases,” and proposed an amendment to the 2004 NIH appropriations bill (which regulates the research funded by NIH). The Amendment was rejected, but more than 200 NIH-funded grants, most of which examined behaviors that affect the spread of HIV/AIDS, were internally reviewed later that year; NIH defended each grant, so none were curtailed. In this study, Joanna Kempner investigates how the scientists whose US federal grants were targeted in this clash between politics and science responded to the political controversy.

**What Did the Researchers Do and Find?:**

Kempner interviewed 30 of the 162 principal investigators (PIs) whose grants were reviewed. She asked them to describe their research, the grants that were reviewed, and their experience with NIH before, during, and after the controversy. She also asked them whether this experience had changed their research practice. She then used the information from these interviews to design a survey that she sent to all the PIs whose grants had been reviewed; 82 responded. About half of the scientists interviewed and/or surveyed reported that they now remove “red flag” words (for example, “AIDS” and “homosexual”) from the titles and abstracts of their grant applications. About one-fourth of the respondents no longer included controversial topics (for example, “abortion” and “emergency contraception”) in their research agendas, and four researchers had made major career changes as a result of the controversy. Finally, about 10% of respondents said that their experience had strengthened their commitment to see their research completed and its results published although even many of these scientists also engaged in some self-censorship.

**What Do These Findings Mean?:**

These findings show that, even though no funding was withdrawn, self-censoring is now common among the scientists whose grants were targeted during this particular political controversy. Because this study included researchers in only one area of health research, its findings may not be generalizable to other areas of research. Furthermore, because only half of the PIs involved in the controversy responded to the survey, these findings may be affected by selection bias. That is, the scientists most anxious about the effects of political controversy on their research funding (and thus more likely to engage in self-censorship) may not have responded. Nevertheless, these findings suggest that the political environment might have a powerful effect on self-censorship by scientists and might dissuade some scientists from embarking on research projects that they would otherwise have pursued. Further research into what Kempner calls the “chilling effect” of political controversy on scientific research is now needed to ensure that a healthy balance can be struck between political involvement in scientific decision making and scientific progress.

**Additional Information.:**

Please access these Web sites via the online version of this summary at http://dx.doi.org/10.1371/journal.pmed.0050222.

- The Consortium of Social Science Associations, an advocacy organization that provides a bridge between the academic research community and Washington policymakers, has more information about the political controversy initiated by Patrick Toomey
- Some of Kempner's previous research on self-censorship by scientists is described in a 2005 National Geographic news article

## Introduction

A number of reports have claimed that the Bush administration in the United States has suppressed, distorted, and manipulated research failing to support its ideologies and interests [1–4]. Public health research, especially on topics such as abortion, abstinence education, risky sexual practices, condom use, emergency contraception, and sex workers, is at the center of these charges. For example, the US Food and Drug Administration (FDA) overruled its own expert advisory panel's recommendation to approve emergency contraception for over-the-counter use [5]; the US Department of Health and Human Services (DHHS) issued travel quotas, that effectively limited attendance by government scientists at international AIDS conferences [6]; the National Cancer Institute posted a Web site suggesting that a link between abortion and breast cancer might exist despite substantial evidence refuting this connection [7]; and the administrators of POPLINE, a publicly funded database housed at Johns Hopkins University, removed “abortion” as a searchable keyword term in response to concerns from their federal funders [8]. In Congress, the Republican Study Committee has made part of their explicit policy platform the excision of NIH grants funding study topics they deem “unworthy” of federal tax dollars [9].

Scientists, journalists, and media pundits have signaled their concern that these political controversies in science may have a wide-ranging “chilling effect” on the work that researchers choose to undertake [10–14]. But what evidence is there that researchers are in fact engaging in self-censorship?

There is a tendency to think of scientists as sociologist Robert Merton once described them: as members of an intellectual community guided by norms of openness and transparency and committed to critique, organized skepticism, and the production of objective knowledge [15]. But as many science studies scholars have demonstrated, this ideal world poorly describes actual practice [16]. For example, imagining science as a producer of objective knowledge obscures the extent to which science relies on its sponsors for funds [17]. From this perspective, the boundary between science and politics is itself questionable. Scientists must be savvy political actors to create and maintain the resources and networks necessary to produce knowledge [18].

Although a rich body of literature examines how these funding relationships can shape the production of knowledge, scholars have only recently begun to study how these same forces might systematically create pockets of “nonknowledge” (Frickel S, Gibbon S, Howard J, Kempner J, Ottinger G, et al., personal communication) [19]. Most research on censorship in science has examined the direct suppression, distortion, or manipulation of knowledge and the various ways that employers, funders, and other sponsors can intimidate and silence researchers [20–23]. Less studied are the conditions under which scientists may self-censor. These studies have found that on rare occasions, scientists have organized to suppress whole lines of inquiry perceived to be dangerous, as they did during the 1975 Asilomar moratorium on recombinant DNA [24]. More frequently, scientists self-censor for pragmatic reasons. For example, many scientists self-censor rather than publish findings contrary to disciplinary or ideological boundaries [25,26]. They may avoid controversial areas of research altogether, rather than face burdensome regulatory requirements [27]. Some advocacy groups may also intimidate scientists. Animal rights activists, for example, have successfully dissuaded some scientists from using certain kinds of animal models in research [28].

Could the potential to attract political controversy also serve as a disincentive to conduct research? Given how often science and politics clash, scant attention has been paid to how scientists respond to these political controversies. To begin to fill this gap, this study followed a political controversy in which lawmakers and activists questioned the credibility and morality of NIH grants that funded research on sexual topics [29]. In this article, I assess how NIH-funded researchers who were targeted perceive the impact of this controversy on their research. Researchers whose proposed studies had become the focus of public debate were asked to reflect on their experience: How had this controversy changed their research practices?

## Methods

### The Controversy

The controversy began in July 2003, when then Congressional Representative Patrick Toomey, a Pennsylvania Republican, proposed an amendment to the 2004 NIH appropriations bill that would rescind the funding of five NIH grants—four of which examined sexual behavior, including studies of transgendered Native Americans, undocumented Asian sex workers, the sexuality of aging men, and the relation of mood to sexual risk taking [30]. Toomey argued that these studies were “much less worthy of taxpayer funding” than research on “devastating diseases” and asked publicly, “who thinks up this stuff?” [30]. The amendment failed to pass the House by two votes.

In October 2003, before a joint hearing of the House Energy and Commerce Committee and Senate Health, Education, Labor and Pensions Committee, several Republican members of Congress asked NIH Director Elias Zerhouni to explain the “medical benefit” of the five NIH grants included in the original Toomey Amendment, plus an additional five grants. Eight of these ten grants addressed sexual behavior [31]. However, the next day the committee staffer responsible for forwarding the list of grants in question to Zerhouni's office sent the wrong document; instead of ten grants, the NIH received a list of more than 250 grants by 157 principal investigators (PIs) [31]. Most of these grants also investigated sexual behavior and drug use, among other HIV/AIDS-related behaviors. Republicans apologized, calling the distribution of this list accidental, and asked the NIH to ignore it. (Some weeks later, the Traditional Values Coalition, a self-described conservative Christian lobbying group, claimed authorship of this list, hereafter referred to as “the list” [11]). Nevertheless, Zerhouni ordered a review of each NIH grant mentioned [32]. This review concluded that all studies were scientifically sound and in January 2004, Zerhouni wrote to Congress saying, “the constant battle against illness and disease…cannot be limited to biological factors but has to include behavioral and social factors as well” [33].

All grants remained funded, but scientists, journalists, and media pundits expressed concerns that these events would have a “chilling effect” on future scientific research [10–14].

### Data Collection

Two waves of data were collected for the present study. The first wave, collected between October 2005 and June 2006, includes data collected via in-depth interviews with a stratified random sample of 30 PIs named in these controversies. The second wave includes data collected from a survey that was distributed to all PIs involved in these controversies. Eighty-two PIs completed the survey between June and October 2006.

This project received institutional review board approval from the University of Michigan and Princeton University, and all participants gave informed consent prior to participation. Details on the protocol and consent forms can be found in Text S1.

### Interview Study

This study began with in-depth interviews. This method is an appropriate data collection tool to use when little is known about the topic under study, as is the case with the conditions under which scientists choose to self-censor and the variety of ways in which this censorship might be enacted [34]. More than surveys, interviews allow for participants to articulate the reasoning behind their decisions and allow for unexpected findings to emerge.

A stratified random sample was used to increase variation around the following variables: level of involvement in the controversy (i.e., PIs who were named on the Congressional floor were sampled separately from those named on the list that was later circulated in Congress); seniority as a researcher (assistant professors; associate professors; full professors); and type of employer (academic versus research institutions) (Table 1).

**Table 1:**
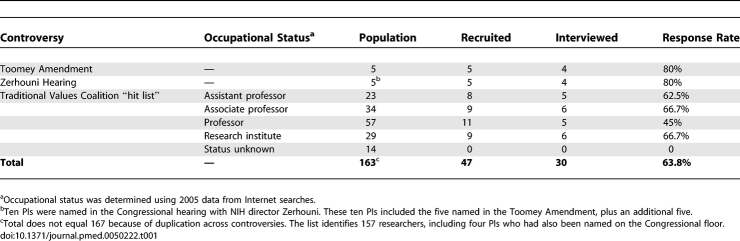
Sample Selection for Interview Data

Eight of the ten researchers whose grants were named on the Congressional floor (either in the Toomey Amendment or during the Zerhouni hearing) agreed to participate. The remaining sample was drawn from the list of 157 researchers (Table 2). A copy of the list was obtained from a scientific interest group. Individual researchers' job titles and places of work were identified via Internet searches and determined using 2005 contact information. Junior faculty and associate faculty were over-sampled, as full professors dominated the list.

**Table 2:**
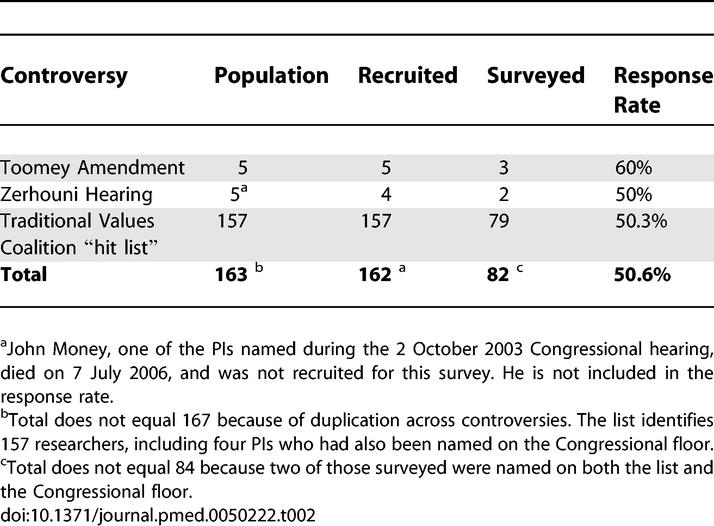
Sample Selection for Survey Data

PIs were initially contacted via a letter written on University of Michigan letterhead. Subsequent requests for interviews were made by email and telephone. Interviews were conducted by telephone, audio-taped, and lasted an average of 64 min. PIs were asked to describe their research, the grant or grants that were reviewed, and their experience with NIH before, during, and after this controversy. PIs also were asked to reflect on how this experience changed their research practices. Questions were open-ended: “How did this controversy change the way that you did research?” and then followed with probes: “For example, has it persuaded you from doing sex-related research?” In addition, researchers were asked whether their work had ever before been the subject of controversy and to discuss their strategies for managing controversies. The interview guide is available upon request.

Interview data underwent two levels of coding: the first level was deductive and captured participants' answers to individual questions posed to them. The second level of coding followed the inductive guidelines set forth in grounded theory. This process allowed the researcher to generate hypotheses and develop theoretical formulations that emerged from participant's experience [35].

### Survey Study

The interviews provided important data about when, why, and how researchers chose to self-censor, but a larger sample was needed to assess the prevalence of these practices. Preliminary analysis from interviews allowed for the construction of a survey instrument [36].

In the second wave of data collection, the survey was distributed to the entire population under study, which included the PIs of five grants named in Toomey's original amendment, the additional five PIs whose grants Zerhouni was asked to justify during the 2 October 2003 Congressional hearing, and 157 PIs on the list. The total population equals 162 because of duplication across the lists (Table 2). Demographic characteristics are listed in Table 3. In total, 82 participants completed the survey for a response rate of 51%.

**Table 3:**
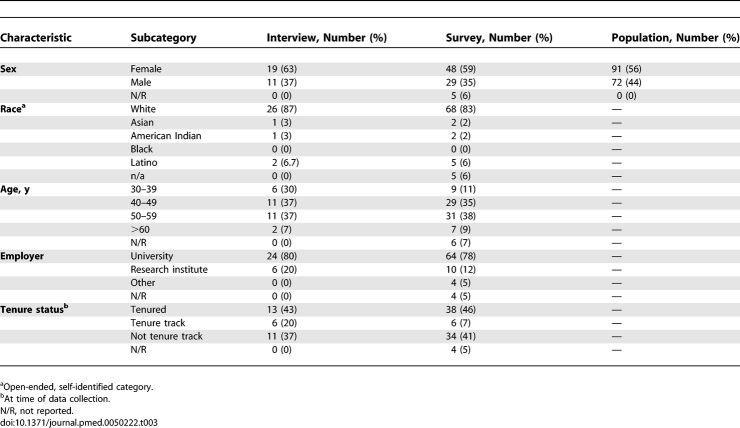
Demographic Characteristics of Interview and Survey Respondents

Using a combination of close-ended and open-ended questions, participants were asked about their experiences with this controversy and how they changed their research practices in response. Close-ended questions produced standardized results that were useful for statistical analyses, while open-ended questions allowed interviewees to elaborate. The survey ended with a series of five attitudinal questions, the results of which are presented in Table 4. The survey was piloted on three participants to test for comprehension and readability. The final survey took about 12 min to complete. The survey (available upon request) was distributed online using an Internet survey software program. Data were analyzed using frequency and cross-tab procedures using SPSS software [37]. In the analysis, data marked (S) are drawn from the sample. Data marked (I) are drawn from interviews. All percentages refer to survey data only.

**Table 4:**
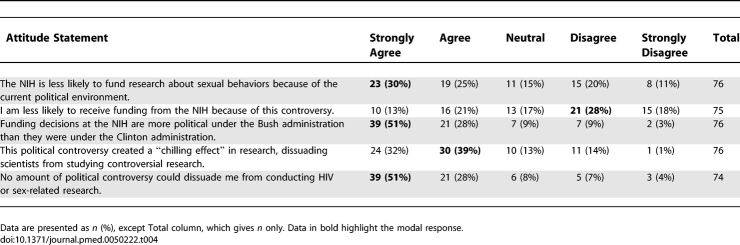
Scientists' Attitudes Regarding the Politics of Sex Research at the NIH (Survey Results)

## Results

As shown in Table 4, the majority of those surveyed described a dramatically changed political environment. Over half either strongly agreed (30%) or agreed (25%) with the statement that “The NIH is less likely to fund research about sexual behaviors because of the current political environment.” Most either strongly agreed (32%) or agreed (39%) that this controversy created a “chilling effect.” A majority (54%) reported feeling nervous, fearful, or even “paranoid” about the political environment. But only one-third strongly agreed (13%) or agreed (21%) that they were less likely to receive funding from the NIH because of this controversy—even if they thought that the NIH was less likely to fund others who applied for grant money to do sex-related research.

At first, these data seem contradictory. Why did some PIs express anxiety, even as they remained confident that their ability to obtain funding would be unhindered? As outlined below, a closer examination of the data revealed that many PIs assuaged their anxieties by engaging in a variety of self-censorship practices. Paradoxically, researchers believed that by strategically self-censoring they could continue to receive funding for sex-related research. These self-censorship strategies existed on a continuum ranging from complete silence at one extreme to minor modifications and omissions at the other.

**Figure 1:**
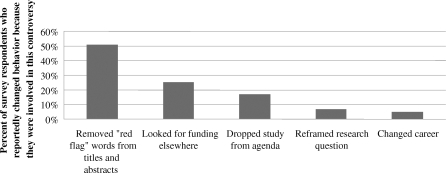
Self-Censorship Strategies in Use by Researchers

Most often, researchers tried to “game” the system by continuing to do their research as before while employing practices specifically designed to disguise the most controversial aspects of their research. Half (51%), for example, said that they removed potential “red flag” words from titles and abstracts of their subsequent NIH grant submissions. Deleted words included: gay; lesbian; bisexual; sexual intercourse; anal sex; homosexual; homophobia; AIDS; bare backing; bathhouses; sex workers; needle-exchange; and harm-reduction. As one interviewee put it: “I do not study sex workers, I study ‘women at risk'” (survey data [S]) (Figure 1).

Removing controversial words from federal grants is not a new strategy for avoiding controversy [38,39]. Nevertheless, most researchers assume that the Traditional Values Coalition compiled their list using a keyword search on CRISP (http:/crisp.cit.nih.gov/), the NIH's public database of grants. Deleting possible keywords on subsequent grant proposals is not only a logical strategy; according to PIs, it is often recommended by NIH project officers. Most PIs described these strategies as cosmetic and inconsequential. Others found these practices more damaging, arguing that minor changes in language obscure the actual content of grants and make it harder to find “cutting-edge grants” in the CRISP database. A few PIs said that project officers' language recommendations sometimes went beyond rephrasing. Depending on their recommendations, they could also be, as one PI claimed, “a question, if you're going to be honest...of doing a different project” (interview data [I]).

For about a quarter of participants (24%), self-censorship extended beyond simple language changes. For some (7%), studies were reframed in ways thought to be less politically sensitive, perhaps by avoiding research on marginalized or stigmatized populations. For example, a sexuality researcher reported that they chose to forego studies on single men and women with minority sexual preferences in favor of studies on the role of sexual health within monogamous, married heterosexual couples. Or abstinence was included in a study even though, as one PI who used this strategy said, it has “been shown to not work” (S). More often (17%), researchers dropped studies or chose not to renew studies that they (or their administrators) believed to be politically nonviable. For example, one researcher described how “we had written a proposal and it had gotten reviewed and we had gotten comments, and it was waiting to be revised and resubmitted. But we kind of sat on that and decided to pursue that a little bit later. And I think that was affected, that decision was very much affected by what was going on…because there was clearly, um, a viewpoint that, you know, that population of MSM [men who have sex with men], for example, was not something that...that should be funded” (I).

Research topics avoided as a result of the controversy included: the sexual health and/or orientation of adolescents; abortion; emergency contraception; condom use; anal sex; childhood sexual abuse; homosexuality; and the use of various harm reduction strategies.

One-fourth (25%) of the respondents said that this controversy made them more likely to seek funding outside of the NIH. Yet all but eight of the survey respondents had already submitted another grant proposal to the NIH for consideration. PIs explained that, in general, they preferred to submit an NIH grant that they believed was politically viable (an act that might require self-censoring) rather than to seek alternative funding from a nongovernmental source. Only federal grants, they explained in interviews, could support the large-scale projects that they were interested in conducting.

Nevertheless, four of 82 PIs reportedly made specific, sometimes dramatic, changes to their careers as a direct result of this controversy. Two left research positions, in which they had to raise their own salaries by securing grant money for the security of research jobs with guaranteed salaries. A third continued their research, but “left the country for a more supportive science environment” (S). The fourth left academic research altogether, declaring that “This [controversy] ended my research career” (S).

It is important to note, however, that this controversy may also have galvanized segments of this research community, thereby increasing social integration in the community. Ten per cent of this sample said they felt “pride” upon learning that their name had appeared on the list. An additional 37% felt “pride” along with anxiety and anger. Several came to see their name's appearance on the list as a “badge of honor” and “motivation to…meet all the objectives of the grant” (I). This group described the list as a racist, sexist document that reflected what participants saw as moral failings of a political leadership gone astray. As one PI put it, “If I am attacked by these people, it's an honor…” (I). It is important to note, however, that even as these researchers argued that these controversies strengthened their commitment to see their research completed and disseminated widely, they, too, engaged in self-censorship practices.

No obvious characteristics (tenure status, seniority, expertise, gender, and discipline) distinguished those who self-censored from those emboldened by this controversy, perhaps because this sample is too small for such differences to emerge as meaningful.

## Discussion

A majority of the researchers reported that their experience of being targeted by Congressional representatives or the Traditional Values Coalition led them to engage in a number of self-censorship practices (Figure 1). Over half “cleansed” grant applications of controversial language, but many also reframed studies, removed research topics from their agendas, and, in a few cases, changed their jobs. Overall, they viewed these actions as important and even necessary strategies to use when applying for federal grants to do potentially controversial research.

This particular controversy should be understood within the context of similar political disputes surrounding the production and dissemination of sex-related research both nationally and abroad. For example, in the same year as the Toomey Amendment/Traditional Values Coalition controversy, the United States Agency for International Development's “Prostitution Pledge” meant that HIV researchers could no longer collaborate with any organization thought to assist sex workers, if they wanted to continue receiving federal funds [40,41]. In addition, the Bush administration not only continued to fund abstinence-only education despite evidence that such programs do not work, but according to a 2004 report released by Congressman Henry A. Waxman, these programs were also disseminating inaccurate and misleading information about the efficacy of contraceptives. And, as mentioned above, the DHHS issued travel quotas that effectively limited attendance by government scientists at international AIDS conferences [6].

That these political controversies act as an “informal constraint” shaping what some researchers choose not to study is not surprising [28]. Applying for grants is a necessary, albeit time-consuming process, complicated by ever-increasing competition for limited federal funds. To be successful, researchers need to intuit funders' desires to shape their research questions into fundable projects. Thus, the operative term “self-censorship” deserves some analytic attention, since the editing of research agendas to fit funders' priorities is an everyday scientific activity that all researchers must engage in as part of fundraising efforts. These silences are always a response to power, but in this case, the threat has been made explicit. This article reserves the term “self-censorship” for silences reportedly generated by political controversies—in this case, the Toomey Amendment and Traditional Values Coalition list. If, for example, a researcher had stopped studying sexuality but said that this shift in focus had been motivated by something other than this controversy, their silence was not counted as self-censorship.

While this methodological approach allows for a study of nonknowledge, it comes with some limitations. Notably, participants may not be reliable narrators of their own experience. Findings may suffer from recall bias, as interviewees were reporting on events that had occurred 2–3 y prior to the study. Some participants mitigated this bias by referring to personal files where they had tracked correspondence related to the controversy. Time may also color PIs' assessments of the controversy's impact on their behavior. In the 3 y that lapsed between the controversy and data collection, researchers had grants submitted, accepted, and rejected. Careers changed, for better and for worse. These events served as a prism through which participants reflected on this controversy and its consequences. As such, this study cannot demonstrate a causal relationship between this political controversy and self-censorship practices.

That any researchers cited this controversy as the reason for dropping out of the academy is remarkable, especially considering that no grant money had been revoked. Moreover, this study accounts only for a segment of the academic community, as the sample was limited to those few researchers already successful enough to serve as PIs on their own NIH grants. The broader implications of political controversy for the research community—for example, funding agencies, graduate students, junior researchers, and other researchers who work in HIV prevention, but who escaped the list—remain unknown.

Selection bias in sampling procedures is also a concern. The response rates fell within an expected range for a survey of professionals (wave 1, 64%; wave 2, 51%). But the substantive topic may have systematically encouraged certain individuals who were eager to speak out, while discouraging others who were too anxious about the potential for political retribution to participate. Reactions to recruitment efforts provide some clues: three potential participants declined explicitly out of fear that these data might be subpoenaed. However, eight PIs expressed genuine enthusiasm about the study. Thus, selection bias is likely to be bidirectional and both underestimate rates of self-censorship and overestimate the potential that this controversy would have the opposite effect and create incentives for researchers to conduct this research.

Unfortunately, researchers' strategy of cleansing titles and abstracts of controversial keywords makes it difficult to assess objectively (for example, via keyword searches on CRISP) whether funding levels have, in fact, dropped for sexuality grants. In other words, Congressional oversight has, in this case, had the unintended consequence of making science less transparent.

This study finds that political environment can serve as a powerful force shaping scientists' research practices: some scientists shy away from controversial research areas, while others relish the opportunity to defend their ideological positions. Public discussion of political involvement in scientific domains has focused on blunt claims: the suppression, distortion, and manipulation of results to further ideological, political and corporate agendas. Focusing debate at this level is important, but it remains to be explored exactly how scientists may self-censor in response to the political environment.

Finally, controversies like the Toomey Amendment and Traditional Values Coalition list serve as an important reminder that federally funded research is subject to Congressional oversight. At the same time, these controversies raise important policy questions about the proper exercise of that oversight. What public interest is that oversight intended to protect? What happens when that oversight encourages scientists to obscure the content of their research? How can science serve the public good, broadly defined, when inquiry that threatens political interests, narrowly defined, is suppressed and that suppression is justified as routine oversight? There is a role for democratic public engagement in science. The policy challenge will be to encourage this public voice in scientific decision-making, while enabling scientists to submit and conduct innovative studies, even when they may provoke controversy.

## Supporting Information

Text S1 Protocols and Consent Forms (408 KB DOC)

## Abbreviations

DHHS: US Department of Health and Human Services
FDA: US Food and Drug Administration
NIH: US National Institutes of Health
PI: principal investigator
