# Correction: The Chilling Effect: How Do Researchers React to Controversy?

**DOI:** 10.1371/journal.pmed.1000014

**Published:** 2009-01-27

**Authors:** Joanna Kempner

Correction for:

Kempner J (2008) The chilling effect: How do researchers react to controversy? PLoS Med 5(11): e222. doi:10.1371/journal.pmed.0050222

An intermediate version of the interview guide was incorrectly included in Text S1 and published in the Supporting Information section. An updated version of the interview guide can be found in the corrected Text S1 below.

**Supporting Information**

Corrected Text S1.

Protocols and Consent Forms

10.1371/journal.pmed.1000014.sd001 (450 KB DOC).

