# Supplementary material for: The Chilling Effect: How Do Researchers React to Controversy?
Source: PLoS Med. 2008 Nov 18;5(11):e222. doi: 10.1371/journal.pmed.0050222 (PMC2586361; doi:10.1371/journal.pmed.0050222)
Supplement: Supplementary file 1 [file pmed.0050222.sd001.doc]

**UNIVERSITY OF MICHIGAN**

## CONSENT FORM

Principal Investigator: Joanna Kempner, Ph.D.

Department of Health Management and Policy

School of Public Health

734-936-1297 (ph)

866-337-3949 (fax)

### **Assessing the effects of political controversy on scientific agendas**

Application #: H05-00008506-I

**INVITATION TO PARTICIPATE**: You are being asked to participate in a research study because one of your NIH research grants appeared on a list of "troublesome" grants funded by the NIH. This is not a partisan study, nor is it sponsored or affiliated with any interest groups. I am an investigator at the University of Michigan and this study is sponsored by the Robert Wood Johnson Foundation.

**PURPOSE**: In 2003, 2004, and 2005, Republican Congressmen attempted to rescind the funding for several independent NIH grants. This study will assess whether these political actions have had an effect on the kinds of research that scientists choose to do. We are interviewing principal investigators like you. We are also interviewing participants in this political process and program officers at the NIH. This research will help policy makers understand whether their actions are producing the intended effects.

**PROCEDURES:** Participation in this study involves a confidential 40-60 minute audio-taped interview. I will not record your name with the tape, and I will destroy all information linking you to your responses as soon as possible. If you choose to participate, I will ask you to reflect on the decisions that you make about research, your involvement with the NIH investigations, your opinions about this controversy, and whether you think that this amendment had an effect on the kinds of studies that researchers are able to do. I will also ask for your opinions about the relationship between government and science.

**Before participating in this study, you should understand that:**

- **Participation in this research is voluntary.**
- **This project is not linked to any other research**
- **This research is not linked to any political organization.**
- **You may refuse to answer any question in the interview.**
- **You may stop the interview at any time.**
- **This interview will be audio-taped.**

**RISKS**: I may ask you to discuss practices that could be damaging to your professional reputation and standing, if disclosed outside of the research. See "Confidentiality" below.

**COSTS AND FINANCIAL RISKS**: There are no costs to you as a participant in this study.

**BENEFITS**: This research will lay the groundwork for a better understanding of how some issues are identified as a problem that should be legislated, while others are not. In addition, this research will assess whether political action has an effect on the kinds of research that scientists choose to do. This research will help legislators understand whether their actions are producing the intended effects.

**COMPENSATION**: There will be no financial compensation for participation.

**CONFIDENTIALITY**: Every attempt will be made by the investigators to maintain all information collected in this study strictly confidential, except as may be required by court order or by law. Authorized representatives of the University of Michigan Institutional Review Board (IRB), a committee charged with protecting the rights and welfare of research subjects, may be provided access to research records that identify you by name. Your name and any other identifying information will be deleted from any publication or presentations that result from this research.

**DISCLAIMER/WITHDRAWAL**: You agree that your participation in this study is completely voluntary and that you may withdraw at any time.

**SUBJECT RIGHTS**: If you wish further information regarding your rights as a research subject, you may contact the University of Michigan Institutional Review Board, Kate Keever, 540 East Liberty Street, Suite 202, Ann Arbor, MI 48104-2210, (734) 936-0933.

Email: irbhsbs@umich.edu

You also understand that if you have any questions pertaining to your participation in this research study or any research related injury, you may contact the principal investigator by calling the telephone number(s) listed at the top of page one.

You have been given the opportunity to ask questions and have had them answered to your satisfaction.

**CONCLUSION**: You have read and understand the consent form. You agree to participate in this research study.

| Name of Subject |  | Signature of Subject |  | Date/Time |
| --- | --- | --- | --- | --- |
|  |  |  |  |  |
| Name of Person Obtaining Consent |  | Signature of Person Obtaining Consent |  | Date/Time |
|  |  |  |  |  |
| Name of Investigator  (if different from above) |  | Signature of Investigator  (if different from above) |  | Date/Time |

**Interview Guide**

**Investigated Principal Investigators**

Investigator ID__________________	Date/Time___________________________

Interviewer**______________________**

**PART 1. INTRODUCTION**

1. *Greeting, e.g. :* Thanks for setting aside some time for this.

[Has the Respondent [R] returned an informed consent form?]

**If YES,** turn on the recorder, thank the R and say:

"Thanks for sending us the informed consent. As a reminder, this phone call is being recorded, is that okay?"

YES: "Great." *[note: R is allowed to ask for the taping to stop at any time, while continuing the interview. At these points (which are probably the most critical), take detailed notes.* Skip to [1.2]

NO: "Are you sure? Recording the interview makes it much easier for me to take notes; it also ensures that I accurately capture your comments." [If still no] Okay, I understand. I may have to pause in order to take notes, so please bear with me."

**IF NO:** "I haven't yet received your consent form. Have you sent it off yet? (if it's on the way, then thank and skip ahead.) Before we start the interview, I'm going to need you to fax me a copy of your consent form. Do you need another copy?"

B. *Informed Consent – Robyn will work on informed consent process*

2. "This is one part of a research study about the politics of funding sex research. We are interviewing principal investigators, who, like you, appeared on a list of research studies that was compiled by the Traditional Values Coalition. Over the next 45 minutes or so, I'm going to ask you to describe your research; and your experiences with NIH and politics over the past couple of years. Do you have any questions before we begin?"

[Answer questions as best you can, without being leading]

**PART 2. BACKGROUND**

*The purpose of this section is to 1) warm up the participant, 2) assess the centrality of sex research to participant’s work, and 3) to assess participant’s rationale for pursuing sex research.*

1. To start off, could you describe the kind of work that you do?

*Probe: And how long have you been doing sex-related research?*

*Probe: What attracted you to these topics?*

How long have you been doing sex-related research?

*What attracted you to these topics?*

*Probe: How did you get interested in this research area?*

*What's your approach? (disciplinary perspective? Survey data?)*

*Where is your primary appointment? Who, would you say is your intended audience (e.g. policy makers, Academics, etc.)?*

*Why do you think this type of research is important?*

**PART 3. TARGETED GRANT**

*In this section of the interview, I want to learn more about the mechanics of writing a grant, howyou decide whether to write a grant, how long it takes, and how many people work on it.*

1. Tell me about the grant(s) that came under attack.

*Probe:* What did you find?

2. How did you come to study this/these particular grant(s)?
3. Can you tell me about the mechanics of putting together a grant like this?

How long does it take you, on average, to write a grant like this?

How many people does it take to write up a grant like this?

When deciding to write an NIH grant, what sorts of things do you take into consideration? E.g., do you first talk to a program officer? Does the amount of money matter? How about the bureaucratic hassle?

Do you think that there are research taboos at the NIH? Things that they just won't fund?

4. Who were your collaborators? How did you find them?

*Probe: are they all people who have worked in this subject area before? Or are some of them new to this area?*

5. Who was the program officers on this project?
6. How did [program officer] help you through the grant writing process?
7. Was your grant written in response to an RFP? (Request for proposals)
8. Is this your first NIH grant?

Probe: Do you ever work with other funders?

**PART 4. PROCESS BETWEEN INVESTIGATON & EXPLANATION SUBMITTAL**

*The purpose of this section is to gather information about the process between being notified that the participants grant was under investigation and the submittal of their grant explanation.*

9. How did you become aware that your grant(s) was part of this controversy? Was this the first time you heard about this list? If not, how did you first hear about the list?

*Probe more for details here.*

10. What happened next? What did the NIH ask for?
11. Was there an investigation? Did you talk to the program officer?
12. Did your collaborators and staff have to write up anything?
13. Did you talk to others who were investigated? When? What was said? Was there a group strategy?

**PART 5. THE CONTROVERSY & BEHAVIOR**

*The purpose of this section is to gather information about how the controversy initially affected the participant and how the respondent and other’s around him/her responded to the controversy. E.g., Was there a chilling effect.*

14. What was your reaction? How did you respond? *(of course, only ask if not asked before).*
15. What was the reaction of people at the University (*support, conflict, avoidance, etc.)*?

*Probes:*

*Colleagues within the department?*

*Department chair?*

*Administration? Did you deal with anyone else in administration? Did the Dean or the lawyers get involved? What about people in public affairs?*

*Dean prepared some sort of testimony to congress – how does she know about this?*

*Other investigators on the list?*

*How did your department chair react? Did the dept chair offer support? What kind?How did she know about the dept. chair?Did they have a meeting where s/he expressed concern?*

16. Were you worried that your grant money might be pulled?
17. Did you ever have the sense that this might hurt your career?

*Probe:* How?

*Probe:* Might it have helped?

18. Several interest groups have made an issue out of this list. Have you been in contact with any of them? Who? What was that contact like?

*e.g., COSSA, Coalition to Protect Research (CPR), American Psychological Association, American Sociological Association?. Etc.*

19. What have your discussions with your program officer since been like?

*Probe: Who is your program officer?*

*Was s/he supportive? How did s/he help you move through the process?*

20. Did you get a sense of the politics internal to NIH?
    - *Probe: For example, the New York Times reported that NIH program officers have been asking people “to cleanse” their titles of anything that might attract unwanted political attention.*
    - *Probe: I've also heard that program officers are sometimes telling prospective investigators that their proposed projects are just too politically controversial at the moment. Have you heard about this? Has it happened to you or someone who you know?*

"Do you know if the NIH does anything to certain grants to lower their profile? (or, something clearer than that.)

21. Did the media contact you? Did you do an interview?

**Behavior good transition!**

22. Did the controversy change the way that you do research?

*e.g., at level of*

*a. Topic?(has it dissuaded you from doing sex-related research?)*

*b. Types of questions?(has it changed the way that you frame your research questions?)*

*c. Types of data collected?( are there certain survey questions you won't include?)*

*d. Analyses?*

*e. Decisions surrounding what or whether to publish?*

*f Is there anything you would not research now?*

"Can you give me an example of [whatever it is that R is being vague about]?"

23. To your knowledge, did the controversy change the way that your colleagues do research?
24. Do you think that this controversy make it less likely for grants like yours to be funded in the future? Why or why not?
25. Do you worry about the future funding of sex-related research more generally?

Why is that?

26. Have you submitted another grant to NIH since this has happened? What about a different fund-granting organization? Are you more likely to submit a grant to another institution?
27. Has anything good come out of this controversy?
28. Did this controversy affect your relationship with your study population?

*Probe*: How?

**PART 7. POLITICAL CONTROVERSIES**

*The purpose of this section is to gather information about the political implications of the grant that was under investigation and the participant’s future research.*

29. Was this the first time that your research was the subject of political controversy? Explain.

30. Had it ever before occurred to you that your research might be politically controversial?
31. Have any other interest groups taken offense at your research?
32. This list implied that someone was worried about the political implications of your research. Do you think of your work as political?

*Probes:*

*If no, policy implications?*

*In an idea world, how would you like policy makers to use your work?*

33. Has your research ever been used in a way that you disagree with?
34. Is there anything you **will not** research or publish because you worry about its political implications?
35. This controversy raises some questions about the public's involvement in matters of science – particularly those funded by tax dollars. How do you think the public ought to be involved in decisions about science?
    - Probe: Which aspects of the scientific process ought to be decided democratically? At the agenda-setting level? Should we have a more transparent review process?
    - Probe: Having been through this controversy, how do you feel about the NIH's policy to provide public access to the CRISP database?

36. Should scientists and researchers be held accountable to the public? How should this happen?

Robyn, I'll have to think of more of these, but the general idea is to get the R to comment on democracy in science --

**PART 8. DEMOGRAPHICS**

*Now in this section I would like just a little information from you:*

36. What discipline were you trained in? ___________________________________
37. What department/school are you currently affiliated with? ______________________
38. What is your job title? __________________________________________________
39. How many years have you worked at this job? ___________________________
40. If assistant professor: When will you go up for tenure? ______________________
41. What is your age? __________
42. What is your gender? ___________
43. How would you describe your race/ethnicity? ________________
44. What is your party affiliation?________________________________

**PART 9. CLOSE**

45. That’s all the questions I have for now. We’re in the beginning of this research, and some unexpected questions might come up. Would you mind if we contacted you in the future?
46. Is there anything else you want to add about your experience with this controversy?
47. Is there any way we could improve this interview?
48. Would you like to see the results of the study once it’s complete?

 YES	 NO

**If Yes:** Great. When it's published, we'll send it to you by email. (Get email address if we don't have it.

Email address: ____________________________________________

Thanks. We really appreciate you taking the time to help us out.

**SURVEY**

**(includes informed consent)**

**1. Politics and the NIH**

In late 2003, a few Republican congressmen, in conjunction with the Traditional Values Coalition, raised questions about the legitimacy of over 200 NIH-funded grants. In response, NIH director Elias Zerhouni, ordered an "internal review" of these grants.

We have sent you this survey because your grant(s) appeared on this "hit-list."

The following survey will ask you about the grant(s) that was targeted and how you learned about the existence of this list. It will also ask you to reflect on whether and how this controversy changed the way that you do research. The survey also includes basic demographic information. The survey should take about 10-15 minutes to complete.

Questions can be directed to Joanna Kempner, Ph.D. at jkempner@umich.edu or (734) 936-1297,

The results will be published in peer-reviewed academic journals.

1. If you are interested in receiving notice when these results are published (whether or not you choose to participate), please enter your email address here. Your identity and email address will remain confidential:

**2. Informed Consent**

This is the informed consent. Please read this page carefully and, if you choose to participate, indicate your consent at the bottom of this page. Thank you.


UNIVERSITY OF MICHIGAN
CONSENT FORM

Principal Investigator: Joanna Kempner, Ph.D.
Department of Health Management and Policy
School of Public Health
734-936-1297 (ph)
jkempner@umich.edu

Assessing the effects of political controversy on scientific agendas
Application #: H05-00008506-I

INVITATION TO PARTICIPATE: You are being asked to participate in a research study because one of your NIH research grants appeared on a list of "troublesome" grants funded by the NIH. This is not a partisan study, nor is it sponsored or affiliated with any interest groups. I am an investigator at the University of Michigan and this study is sponsored by the Robert Wood Johnson Foundation.

PROCEDURES: Participation involves the completion of the following confidential survey. I will destroy all information linking any identifying information to your responses as soon as possible. The survey will ask you to reflect on your involvement with the NIH investigations, your opinions about this controversy, and whether you think that this amendment had an effect on the kinds of studies that researchers are able to do.

RISKS: The only risk is a breach of confidentiality. See "Confidentiality" below.

COSTS AND FINANCIAL RISKS: There are no costs to you as a participant in this study.

BENEFITS: There is no direct benefit to you. This research will assess whether political action has an effect on the kinds of research that scientists choose to do. This research will help legislators, NIH officials, and university administrators learn how to better manage certain forms of scientific controversy.

COMPENSATION: There will be no financial compensation for participation.

CONFIDENTIALITY: Every attempt will be made by the investigators to maintain all information collected in this study strictly confidential, except as may be required by court order or by law. Authorized representatives of the University of Michigan Institutional Review Board (IRB), a committee charged with protecting the rights and welfare of research subjects, may be provided access to research records that identify you by name. Your name and any other identifying information will be deleted from any publication or presentations that result from this research.

DISCLAIMER/WITHDRAWAL: You agree that your participation in this study is completely voluntary and that you may withdraw at any time.

SUBJECT RIGHTS: If you wish further information regarding your rights as a research subject, you may contact the University of Michigan Institutional Review Board, Kate Keever, 540 East Liberty Street, Suite 202, Ann Arbor, MI 48104-2210, (734) 936-0933.
Email: irbhsbs@umich.edu

You also understand that if you have any questions pertaining to your participation in this research study or any research related injury, you may contact the principal investigator by calling the telephone number(s) listed at the top of page one.

2. Please indicate that you understand and agree to the following:

|  | Participation in this research is voluntary. |
| --- | --- |
|  | Participation in this research is confidential. |
|  | This research is not linked to any political organization. |
|  | You may refuse to answer any question in the survey. |
|  | You may stop the survey at any time. |

3. You have read and understand the consent form. You agree to participate in this research study.

|  | Yes |
| --- | --- |

|  | No |
| --- | --- |

**Part 4 (of 8)**

The Hit-List

| 10. How did you first learn that your name appeared on this hit-list? | |
| --- | --- |
|  | -An NIH official called/emailed me. |
|  | -A colleague told me about it |
|  | -My university informed me |
|  | -A journalist called me to comment |
|  | -I read about it in the news and checked to see if my name was there |
|  | -I didn't know I was on this list until I received this survey. |
|  | -I don't remember |
|  | -Other (please specify) |
|  |  |

|  | 11. Did anyone from the NIH ever communicate with you about this hit-list? \|;  \| -Yes;  \| -No;  \| -I don't remember |
| --- | --- |
|  | 12. When the hit list was made public in 2003, what was the funding status on your grant?  \| -Closed. I had already received all my NIH funding.;  \| -About to close. I had received most of my NIH funding.;  \| -Ongoing. My grant (or at least one of the grants) was still open.;  \| -Other (please specify) |

13. Were you in contact with any of the other researchers on this hit-list?

|  | -Yes |
| --- | --- |
|  | -No |
|  | -I don't know |

14. Did other researchers at your institution appear on the hit-list?

|  | -Yes |
| --- | --- |
| - | No |
|  | -I don't know |

15. What was your first reaction to learning that your grant appeared on this hit-list? (choose as many as apply)

|  | -Pride |
| --- | --- |
|  | -Anger |
|  | -Nervousness |
|  | -Fear |
|  | -Paranoia (that I was being watched) |
|  | -Other (please specify) |
|  |  |

|  | 16. What are your current feelings about the controversy? - \| Pride; - \| Anger; - \| Nervousness; - \| Fear; - \| Paranoia (that I was being watched); - \| Other (please specify) |
| --- | --- |
|  | 17. Did you think that the NIH might pull your funding?  \| Yes, definitely;  \| I thought it was a strong possibility;  \| I thought it was possible, but unlikely;  \| Not at all worried |

**5. Part 5 (of 8)**

Institutional Supports

|  | 18. Was your university/institution aware that your name appeared on this list?  \| Yes;  \| No;  \| Don't know |
| --- | --- |
|  | 19. How supportive was your university/institution? ( \| Excellent) \| ( \| Good) \| ( \| Sufficient) \| ( \| Poor) \| ( \| Terrible) \| ( \| Not applicable) |
|  | 20. Did your colleagues know that your name was on the hit-list?  \| Yes;  \| No;  \| I don't know |

|  | 21. How supportive were your colleagues? ( \| Excellent) \| ( \| Good) \| ( \| Sufficient) \| ( \| Poor) \| ( \| Terrible) \| ( \| Not aplicable) |
| --- | --- |
|  | 22. How satisfied were you with NIH's response to this hitlist? ( \| Extremely satisfied) \| ( \| Satisfied) \| ( \| Ambivalent) \| ( \| Unsatisfied) \| ( \| Extremely unsatisfied) \| ( \| Not applicable) |

23. Why?

**6. Part 6 (of 8)**

Research practices: The following questions ask you to reflect on whether this controversy changed how your practice research.

24. Are you more or less likely to seek funding from the NIH because of this political controversy?

|  | More likely |
| --- | --- |
|  | Less likely |
|  | Same |
|  | Don't know |
|  | Other (please specify) |
|  |  |

25. Are you more or less likely to seek funding from other sources because of this controversy?

|  | More likely |
| --- | --- |
|  | Less likely |
|  | Same |
|  | Don't know |
|  | Other (please specify) |
|  |  |

|  | 26. Are there research questions or grant proposals that you have chosen not to pursue as a result of this hit-list?  \| No;  \| If yes, please explain |
| --- | --- |
|  | 27. Have you changed the way that you write grant proposals because your name appeared on this list? \| No; \| If yes, please explain |
|  | 28. Some researchers have reportedly deleted potentially controversial words from the titles and abstracts of subsequent NIH grants. Have you done so? \|; \| No, I've never heard of this strategy; \| No, I've heard about it or thought about, but I have not done so.; \| Yes, explain |

| 29. Are you more vigilant in your research methods as a result of this hit-list? (For example, do you now take steps to ensure that your grant is "audit-proof"?) | |
| --- | --- |
|  | Yes |
|  | No |
|  | Sometimes |
|  | Other (please specify) |
|  |  |

| 30. Has this controversy dissuaded you from conducting certain kinds of research? (For example, some researchers have said that they are no longer willing to conduct studies on especially stigmatizing behaviors; others have dropped grant proposals that seemed particularly controversial). | |
| --- | --- |
|  | No |
|  | Yes, explain |
|  |  |

31. Did this controversy make you more careful about what you publish? How?

32. Are there any other ways in which this incident affected the way you do your research?

**7. Part 7 (of 8)**

Subsequent NIH grants

33. How many of your grants have been under review at the NIH since the hit-list emerged in 2003? (include all grants whether or not you are the principal investigator)

34. For how many of these grants are you the principal investigator?

35. Have any of these grants been accepted?

|  | No |
| --- | --- |
|  | Yes |
|  | Not applicable |

36. Have any of these grants received a "revise & resubmit"?

|  | Yes |
| --- | --- |
|  | No |
|  | I don't know |

37. Have any of these grants been rejected?

|  | Yes |
| --- | --- |
|  | No |
|  | Not applicable |

38. Please indicate your level of agreement with the following statements

|  | Strongly agree | Agree | Neutral | Disagree | Strongly disagree | N/A |
| --- | --- | --- | --- | --- | --- | --- |
| The NIH is less likely to fund research about sexual behaviors because of the current political environment. |  |  |  |  |  |  |
| I am less likely to receive funding from the NIH because of this controversy. |  |  |  |  |  |  |
| Funding decisions at the NIH are more political under the Bush administration than they were under the Clinton administration. |  |  |  |  |  |  |
| This political controversy created a "chilling effect" in research, dissuading scientists from studying controversial research. |  |  |  |  |  |  |
| No amount of political controversy could dissuade me from conducting HIV or sex-related research. |  |  |  |  |  |  |
|  |  |  |  |  |  |  |

**8. Part 8 (of 8)**

You're just about finished! We just have a few demographic questions.

39. What is your gender?

| 40. How do you describe your race/ethnicity? | |
| --- | --- |
|  |  |

| 41. How old are you? | |
| --- | --- |
|  |  |

42. Where do you work?

|  | University |
| --- | --- |
|  | Research Institute |
|  | Other (please specify) |
|  |  |

43. How many years have your worked at your current job?

| 44. How is your salary structured? | |
| --- | --- |
|  | My salary is guaranteed (hard money) |
|  | My salary depends on research grants (soft money) |
|  | My salary is a combination of hard and soft money |
|  | Other (please specify) |
|  |  |

| 45. Do you have tenure? | |
| --- | --- |
|  | Yes |
|  | No, but I am in a tenure-track position |
|  | No, I am not in a tenure-track position |
|  | Other (please specify) |
|  |  |

46. What is your political party affiliation?

**9. THANK YOU!**

Thanks for completing this survey.

47. If you would like to receive the results of this study, please enter your email address here. Your identity and email address will remain confidential.

48. Please enter any additional comments or concerns here:
